# Quantum Secure Group Communication

**DOI:** 10.1038/s41598-018-21743-w

**Published:** 2018-03-01

**Authors:** Zheng-Hong Li, M. Suhail Zubairy, M. Al-Amri

**Affiliations:** 1 Department of Physics, Shanghai University, Shanghai, 200444 China; 2 Institute for Quantum Science and Engineering (IQSE) and Department of Physics and Astronomy, Texas A&M University, College Station, Texas 77843-4242 USA; 3 The National Center for Applied Physics, KACST, P.O. Box 6086, Riyadh, 11442 Saudi Arabia; 4 Department of Physics, KKU, P.O. Box 9004, Abha, 61413 Saudi Arabia

## Abstract

We propose a quantum secure group communication protocol for the purpose of sharing the same message among multiple authorized users. Our protocol can remove the need for key management that is needed for the quantum network built on quantum key distribution. Comparing with the secure quantum network based on BB84, we show our protocol is more efficient and securer. Particularly, in the security analysis, we introduce a new way of attack, i.e., the counterfactual quantum attack, which can steal information by “invisible” photons. This invisible photon can reveal a single-photon detector in the photon path without triggering the detector. Moreover, the photon can identify phase operations applied to itself, thereby stealing information. To defeat this counterfactual quantum attack, we propose a quantum multi-user authorization system. It allows us to precisely control the communication time so that the attack can not be completed in time.

## Introduction

An arbitrary unknown quantum state can not be cloned. The statement known as quantum no-cloning theorem^1^ indicates a robust way to secure communication. Based on this, the first quantum key distribution protocol (QKD), BB84^2,3^, is published in 1984. It allows two communicators to generate a unique key to encrypt messages. After that, during three decades of intense research, a mass of quantum secure communication protocols have been designed and published. They include not only QKD protocols^4,5^, but also direct secure quantum communication protocols^6–8^, quantum public-key cryptography^9–12^ and so on^13–17^. In addition, aimed at practical application, techniques such as decoy states^14,18–20^, device independent QKD^21–24^ are also studied.

No doubt, to achieve a quantum secure network is one of the most important goals of all of the above studies^25^, where QKD is the most promising protocol for application. However, considering network environment, QKD has disadvantages. For security reasons, the distributed key in QKD is disposable, which is called one time pad. This brings in the key management problem when more than two communicators are involved^9^. Since all keys are used once and discarded, it is meaningless to share them among communicators for further use. When the number of communicators increases, a mass of keys need to be managed, which takes lots of resources^9^.

A solution to the key management problem in quantum secure network is quantum public-key cryptography^9–12^, which utilizes quantum one-way function^26,27^. Generally speaking, there is a public key that is only capable of encoding message, while there is another private key, which is just for decoding message. As a result, a receiver who holds the private key can collect information from a large number of senders. Thus, unidirectional group to point communication is achieved.

In addition to quantum public-key cryptography, there are multi-party quantum cryptography protocols^28–32^ based on multi-party entanglement states. Those protocols require particles held by different communicators are entangled before the communication. Then, after the communicators perform appropriate measurements (disentanglement process) and negotiate with each other, a shared key can be determined.

In this paper, however, we solve the key management problem by another way. Without utilizing multi-party entanglement states, we create and share a key among more than two users, so that all authorized communicators can use the shared key to encode and decode information. More specifically, this shared key is pre-selected by Bob himself (the key initiator). The key generation process is irrelevant to other communicators (participants) and can be achieved by a quantum random number generator^33^. After that, the key is sent directly and independently to other communicators. Our protocol is based on the Ping-Pong protocol^6^, which is one kind of direct secure quantum communication protocol between two communicators. In the Ping-Pong protocol, Alice (the message receiver) prepares two entangled photons and delivers one of them to Bob (the message sender). At Bob’s end, he can either measure Alice’s photon to check the security (control mode) or operate the photon phase to encode information (message mode). In message mode, Alice collects the operated photon and performs a joint measurement on two photons. By doing so, Alice gets Bob’s information directly. Apparently, Bob can operate many incoming photons from different communicators simultaneously so that he can broadcast the message to all communicators. However, the question to ask is whether the shared key is secure? In the Ping-Pong protocol, the communication security is guaranteed by random check of the entanglement between two photons. This strategy has been discussed and strengthened^34^. In this paper, we do not intend to repeat the discussion but focus on a new attack, the counterfactual quantum attack, which is based on counterfactual quantum communication protocols^35–40^. In ref.^37^, it shows that a phase operation can be traced by an “invisible” photon. More importantly, this “invisible” photon can reveal a single-photon detector in the photon path without alerting the communication system^35^. Based on the above results, we show that it is possible for an eavesdropper, Eve, to steal Bob’s information without being exposed in the Ping-Pong protocol. To defeat this counterfactual quantum attack, we propose a quantum multi-user authorization system. It works because of spatial relativity^41^ and the fact that photon paths in a Michelson interferometer are untraceable. With the quantum multi-user authorization system, we can achieve a quantum secure group communication that allows secure messages to be shared among multiple authorized users.

In the following, there are five sections. In Section II, we present a detailed setup of our protocol. In Section III, we introduce the counterfactual quantum attack. In Section IV, we elaborate on our security strategy, which can verify the identities of all communicators. In the same section, we summarize the procedures of our secure group communication protocol. In section V, we compare our group communication protocol with that based on BB84. We show that our protocol is more efficient and securer since it can deliver a pre-prepared key securely and directly. In Section VI, we present concluding remarks. In addition, we have three supplementaries. In Supplementary I, we discuss the influence of implement imperfection on the group communication. In Supplementary II, we discuss the influence of the imperfection of the transmission channel. In Supplementary III, we show that successful single-cycle counterfactual quantum attack does not exist.

## The proposed setup of a quantum secure group communication

**Figure 1:**
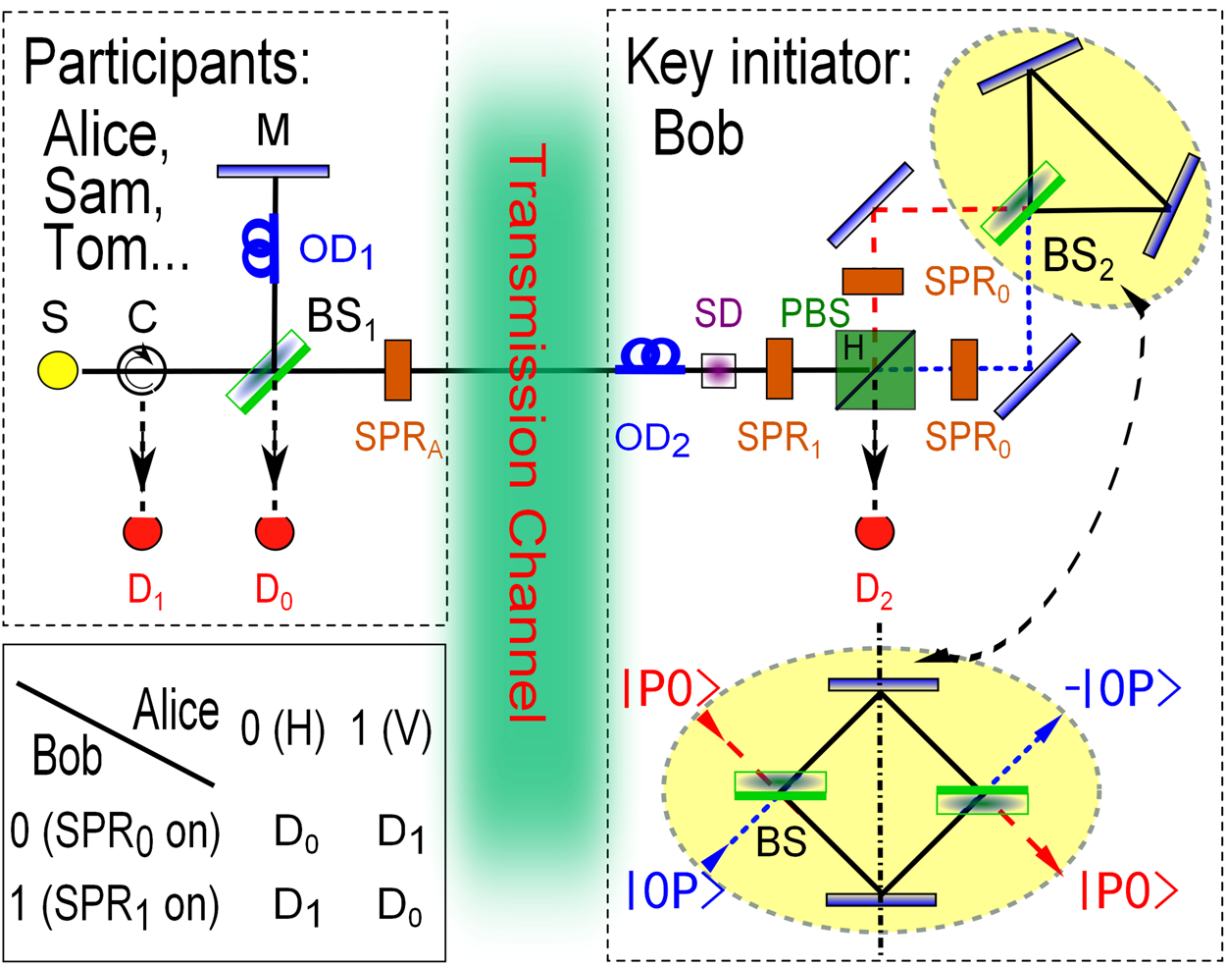
Schematics of the proposed group secure direct communication protocol. In the figure, every participant has the same device which is a Michelson interferometer where *S* stands for light source, *D* stands for photon detector, *C* stands for optical circulator, *BS* stands for beam splitter, *OD* stands for optical delay and *SPR* stands for switchable polarization rotator. In the communication, a participant prepares a horizontal (H) polarized photon for his logic 0 while a vertical (V) polarized photon for his logic 1. After entering the interferometer, the participant’s photon has half the chance of passing through the public transmission channel and reaching the key initiator’s station. To prevent information leakage, *SPR*_*A*_ is randomly activated which can change the polarization of photons from V(H) to H(−V). Thus, in the transmission channel, the photon polarization and the signal of the participant are no longer relevant. At the key initiator’s station, *PBS* stands for polarization beam splitter which reflects only H photon and *SD* stands for switchable detector. *SD* and *OD*_2_ constitute the quantum multi-user authorization system which is used to isolate the key initiator’s device from external environment and to verify the authorization of each incoming photon. For the rest of the key initiator’s device, its function is to operate the photon phase by turning on either *SPR*_0_ or *SPR*_1_. After the phase operation, the key initiator sends the photon back to the participant who then do the measurement. All possible results have been shown in the table.

The proposed setup of a quantum secure group communication is sketched in Fig. 1. Basically, Bob is the key initiator. He continuously broadcasts his signals, which are determined only by him and used as a shared key in the group communication, by operating photons from other communication participants such as Alice, Sam and Tom. All participants’ identities are verified by a multi-user authorization device, which is composed of an optical delay *OD*_2_ and a switchable detector *SD*. Before the discussion of the multi-user authorization system, we first talk about how to achieve information exchange among communicators.

At each participant’s end, there is a Michelson interferometer. As shown in the figure, *C* stands for optical circulator, *D* stands for photon detector, *M* stands for mirror (We assume that all mirrors have no influence on photon phase) and *S* stands for light source, which can generate horizontal (H) polarized photons and vertical (V) polarized photons. Besides that, *SPR* stands for switchable polarization rotator^35^. It is utilized to change photon polarization from V(H) to H(−V) when it is turned on. In addition, *BS* stands for beam splitter with the same transitivity and reflectivity. Here, we point out that the two interfaces of the *BS* are asymmetric (see Fig. 1). Only the reflection at one of the interfaces causes *π* phase shift (Half-wave loss), while transmission and reflection at the other interface do not. Then, the function of the *BS* can be written as^42,43^

1 $$\begin{array}{l}|P0\rangle \to (|P0\rangle +|0P\rangle )/\sqrt{2},\\ |0P\rangle \to (|P0\rangle -|0P\rangle )/\sqrt{2}.\end{array}$$

where *P* = *H*, *V* describes the photon polarization, |0*P*〉 represents that a photon is on the side of the interface with half-wave loss while |*P*0〉 represents a photon is on the other side.

In the communication, a H photon represents participant’s logic 0 while a V photon represents logic 1. After one participant decides his signal, he sends his photon into his interferometer. Due to *BS*, the photon is separated into two paths. One is a private path (between *BS*_1_ and *M*), which is unaccessible to other communicators or eavesdroppers. The other path is a public path which includes an open area (the public transmission channel in Fig. 1) and Bob’s station. Accordingly, the photon state can be represented as $(|P0\rangle +|0P\rangle )/\sqrt{2}$. The photon in the state |*P*0〉 is retained in the private path while the photon in the state |0*P*〉 is in the public path. We notice that $(|H0\rangle +|0H\rangle )/\sqrt{2}$ and $(|V0\rangle +|0V\rangle )/\sqrt{2}$ are orthogonal. By measuring the polarization of the photon in the transmission channel, Eve has 50% chance getting the participant’s information. Therefore, it is unsafe for the participant to launch his photon directly into public path. To prevent information leakage, we add *SPR*_*A*_, which is randomly turned on or off for each participant’s signal. As a result, the polarization of the photon in the open area is no longer consistent with the participant’s information. However, here we should also mention that Eve can not distinguish the above two orthogonal states $(|H0\rangle +|0H\rangle )/\sqrt{2}$ and $(|V0\rangle +|0V\rangle )/\sqrt{2}$ without disturbing them. This is because Eve can only access the public path^15^.

Now the photon component |0*P*〉 is safe and ready to be operated by Bob. Before the discussion of Bob’s operations, here we emphasize that the physical distances between Bob and participants are different. Thus, those optical delays *OD*_1_, which are used to compensate for optical distance difference in participants’ interferometers, are different for different participants.

In light of ref.^6^, Bob’s information can be directly transferred by controlling the phase of participant’s photon. Here, the phase operation is achieved by a polarization beam splitter (*PBS*) reflecting H photon, and an interferometer which is composed of *BS*_2_ and two mirrors. This interferometer is equivalent to a Mach-Zehnder interferometer which is shown in the dotted oval shape at right-bottom of Fig. 1 as well. According to Eq. (1), it is easy to get that the photon coming from the top side (|*P*0〉) must appear eventually at the bottom side without phase difference. We mark the photon path by red dashed lines. In contrary, if a photon is launched from the bottom side (|0*P*〉), it eventually appears at the top side with a *π* phase shift. We mark the photon path by blue dotted lines. Apparently, by selecting the entrance of the incident photon, Bob can control the phase of the photon. This allows Bob to send signals to all participants. In detail, if Bob wants to send a logic 0, he turns off *SPR*_1_ but turns on *SPR*_0_s so that the H photon will be sent into the red path while the V photon will be sent into the blue path. If Bob wants to send a logic 1, he turns *SPR*_0_s off but turns *SPR*_1_ on. Then, the H photon is sent into the blue path while the V photon is sent into the red path.

In the table of Fig. 1, we have shown how one participant can distinguish Bob’s two signals. First we consider one participant turns his *SPR*_*A*_ off (transparent) and sends a H photon to Bob. If Bob encodes ‘0’, *SPR*_1_ doesn’t work. The photon is reflected by *PBS*. Then, it becomes −V due to *SPR*_0_ and goes into Bob’s interferometer by the red dashed path. The phase of its output state does not change. The photon comes back via red dashed path and becomes −H due to *SPR*_0_. Then, the photon goes back to the participant’s place with a *π* phase shift. According to Eq. (1), we have |0*H*〉. The detector *D*_0_ clicks.

If Bob encodes ‘1’, the photon becomes −V according to *SPR*_1_ and then passes through *PBS*. Since it passes through Bob’s interferometer by the blue dotted path, a *π* phase shift appears. According to *SPR*_1_, a H photon goes back to the participant but with a zero phase difference compared to the photon component in the participant’s arm. According to Eq. (1), now we have |*H*0〉, which in turn causes *D*_1_ to click. Here, we should emphasize that, whatever Bob’s decision is (0 or 1), the participant’s photon passes through the active *SPR*s twice and inactive *SPR*s twice. This guarantees that the optical distances in the two cases are the same.

In the above cases, one participant distinguishes Bob’s signals directly by his detectors *D*_0_ and *D*_1_, which achieves a one-way communication. This result is similar to the Ping-Pong protocol but utilizes photon path entanglement instead of two-photon entanglement.

Next we consider the case that the participant still turns *SPR*_*A*_ off but sends a V photon (logic ‘1’). It is easy to find out that *D*_1_ clicks for Bob’s logic 0 while *D*_0_ clicks for Bob’s logic 1. Therefore, in case *SPR*_*A*_ is off, *D*_0_ clicks if the participant and Bob encode the same signal while if they encode different signals, *D*_1_ clicks. Now we look into the case when the participant turns *SPR*_*A*_ on. In this situation, a participant’s photon has an additional *π* phase shift since it passes through *SPR*_*A*_ twice. Then, we shall still see that *D*_1_ clicks if the participant and Bob encode different signals while *D*_0_ clicks if they encode the same signal (see the table in Fig. 1). Subsequently, once the participant publishes his measurement results (which detector of his clicks), Bob knows his messages, and a two-way communication is achieved. Moreover, if Bob operates all participants’ photons simultaneously for his every signal, he can deliver his signals to all participants. With Bob’s signals, any two participants can read each other’s information. A group communication is achieved.

## The counterfactual quantum attack

So far, we have seen how Bob sends a key directly to a group of communicators and how they exchange information based on that key. We note that in addition to multi-user participation, the difference between our protocol and the Ping-Pong protocol is that the polarization of photons transmitted by one participant is not unique. In the previous section, we have shown that the polarization of the photon in the transmission channel does not represent the actual information. Moreover, any detection of photons causes detectable disturbances. Therefore, we can continue to use the security strategy proposed in the Ping-Pong protocol as long as it is not flawed. In the Ping-Pong protocol, the security is ensured by control mode in which Bob randomly stops the message transfer process (message mode) and uses a detector to measure the incoming photon. His measurement result should be related to Alice’s due to entanglement. However, the above security strategy is based on one assumption, i.e., there is no “invisible” photon that does not trigger Bob’s single photon detector but is capable of reading Bob’s phase operation. Unfortunately, according to current research results, this assumption is not true, even if Bob’s detector can detect electromagnetic waves at any frequency.

In ref.^37^, a communication protocol utilizing invisible photons is discussed. It shows how one communicator, Alice, tells if Bob has applied a *π* phase shift to her “invisible” photon by double chained Mach-Zehnder interferometers. If Bob adds a *π* phase shift, Alice’s first detector clicks with unit probability. If Bob decides not to change the phase, Alice’s second detector clicks with unit probability. Then, Alice can collect information from Bob. During the communication, Alice’s photon is sent to Bob several times during his certain operation, but each time the probability of the photon being found is extremely low. More importantly, if Bob continues to observe Alice’s photon instead of manipulating its phase, then the communication becomes a direct counterfactual quantum communication^35^. According to interaction free measurement^44,45^ and Quantum Zeno effect^46–48^, the continuous observation prevents Alice’s photon from leaking into the transmission channel. If Bob does not find Alice’s photon, the photon must locate in Alice’s device and cause Alice’s second detector clicking. Thus, Bob can not see the photon but the photon can sense whether Bob is looking at it. This is counterfactual^35,49^. If unfortunately Bob captures Alice’s photon, the communication failed. However, as we pointed out in ref.^35^, the probability of Bob finding the photon depends on how many times (cycles) that Alice’s photon is sent to Bob. With the increase in the number of times, the probability is close to zero.

Above we briefly introduce how to use an “invisible” photon to do communication, which also implies a method of invisible quantum measurement. Eve can use the method to attack the Ping-Pong protocol without intercepting the message receiver’s photons. Specifically, utilizing the same device proposed in ref.^37^, Eve shoots her own photon towards Bob to do the measurement. She needs to complete a measurement before Bob changes his operation, whether the operation is in message mode or control mode. If Bob selects message mode, Eve definitely can obtain Bob’s information. If Bob selects control mode, Eve’s photon has a tiny probability of being found, which causes her exposure. But the bigger chance is that Bob does not find Eve’s photon, and Eve’s one detector clicks. We note that in control mode, Bob exchanges measurement results with Alice, hence, Eve knows that detector clicking does not represent Bob’s information. As a result, Eve steals Bob’s message. Since the attack is based on direct counterfactual quantum communication protocol, we call it counterfactual quantum attack.

Consequently, the Ping-Pong protocol is not secure due to the counterfactual quantum attack. In the next section, we will present a defense scheme. It works because that a counterfactual quantum attack requires a photon to be bounced between Eve and Bob more than once, which is proved in Supplementary III. Using this feature, we utilize an optical delay system so that Eve is impossible to complete a counterfactual measurement of one Bob’s signal in time. Based on our scheme, the secure strategy in the Ping-Pong protocol works again, i.e., authorized communicators can use single photon detectors to check the entanglement.

## Quantum multi-user authorization system

In this section, we outline and discuss a new approach for checking authorizations of all communications. This method guarantees Bob’s message is only read by the right person. The corresponding device is called the quantum multi-user authorization system, which is made up of *OD*_2_ and *SD* as shown in Fig. 1. In detail, *SD* is controlled by Alice or other participants via public classical channel. The corresponding signal is classical and public. We call it control signal. When *SD* is switched on, it becomes a single photon detector and blocks the path into Bob’s interferometer. If *SD* is off, it becomes transparent for a short time Δ*t*. In this time window, a photon can only pass through Bob’s interferometer once. According to Supplementary III, it is not sufficient to complete a counterfactual measurement. Before *SD*, there is *OD*_2_. We stress that *OD*_2_ is located inside Bob’s station. It is the only way (the quantum channel) for any photon to pass *SD* and enter Bob’s interferometer. Assume the time it takes for a photon to pass through *OD*_2_ is *τ*. Then, in oder to ensure that participants’ photons can pass through *SD* in time, the launch time of the corresponding control signals should be delayed by time *τ*^41^ (For the sake of convenience, we assume that the transmission paths between participants and Bob are straight lines).

Obviously, all participants can get Bob’s information by controlling *SD*, which is their privilege. However, if someone like Eve who is not authorized but wants to get Bob’s signals directly, she needs to know the time window. Even if she wants to implement counterfactual quantum attacks, the information of the time window is still necessary. In order to get the information, Eve can listen to the control signal or measure participants’ photons. Firstly, we consider the situation that Eve carries out the attack based on the control signal. Suppose that Eve immediately starts her attack once she hears a control signal and it takes time *T* for a photon traveling from Eve to Bob. Then, the time required for Eve’s photon to reach *SD* is *T* + *τ*. However, *SD* is transparent from *T* to *T* + Δ*t*. Thus if $\tau \gg {\rm{\Delta }}t$, it is impossible for Eve’s photon to get into Bob’s interferometer. Secondly, we consider the situation that Eve detects participants’ photons instead of listening to control signals. Here we notice that all participants’ photons are path-entangled. They have half a chance localized in participants’ devices which are unaccessible to Eve (private path). As a result, Eve’s eavesdropping must be traceable according to the no-cloning theorem of orthogonal states in a composite system^15^, which says that the two orthogonal states can not be distinguished without disturbing the system, if two subsystems (the private path and the public path in our case) are entangled while one of the subsystem is not accessible. Furthermore, we can understand the aforementioned theorem in a simpler way. As long as Eve gets the time information of a photon, it means that Eve knows exactly that the photon is in the transmission channel. The path entanglement of the photon is destroyed. Consequently, the participant’s detection may display an abnormal result^44,45^.

In general, the quantum multi-user authorization system is utilized to isolate Bob’s station from the external environment. It is a security door of Bob’s station. Only authorized photons can pass through it while an unauthorized entry triggers an alarm. This prevents Eve from stealing Bob’s information by an “invisible” photon or using the same device of the participant (Eve doesn’t have the authorization). This also prevents Eve from exploiting the imperfection of Bob’s optical elements to steal information by sending some modulated light pulse into Bob’s station^50,51^. Therefore, the quantum multi-user authorization system can also protect Bob from side channel attacks such as the Trojan-horse attack^50,51^.

In the above, we show that in principle only authorized communicators can read Bob’s message which can be utilized as the shared key. Eve cannot steal information without leaving traces. In order to reveal these traces, participants can send additional photons to Bob in order to check the entanglement as in the Ping-Pong protocol. The detailed communication protocol is as follow.

### The agreements

Bob and *n* − 1 participants reach the following agreements: (a) Bob’s every signal lasts for time *T*_*s*_. During this time, participants need to complete the measurement of the signal; (b) For Bob’s one signal, each participant launches two photons. Bob decides which photon is used to transfer information. Then, the other photon is for security check; (c) To ensure participants’ photons can be operated without any interference, Bob divides *T*_*s*_ equally into (*n* − 1)*l* slots which lasts Δ*t*. He assigns to each participant *l* slots and informs them.

### Distribution of one signal

#### The preparation

Every participant prepares two photons whose initial polarization is determined by their real information. Polarization H represents logic “0” while V represents logic “1”. In the meantime, each participant generates a random number to decide whether *SPR*_*A*_ is turned on or off so that these photons have random polarizations in the transmission channel. At Bob’s end, he prepares two binary random numbers A and B. He operates every participant’s two photons according to these two numbers. Number “0” means he turns *SPR*_0_ on but turns *SPR*_1_ off while number “1” means he turns *SPR*_1_ on but turns *SPR*_0_ off. In order to distinguish the two photons manipulated by Bob, in the following we call them photon A and photon B, respectively. In addition, for each participant, Bob’s order of operations for A and B is different. The order is decided by Bob randomly.

#### Information transfer

Each participant randomly selects two slots to launch photons. After one participant launches his one photon for time *τ*, he makes an announcement in the public channel so that his photon can pass through *SD* successfully. At Bob’s end, Bob operates those two photons in order. Then, those photons are sent back to their participant and measured. If the participant and Bob encode the same signal, *D*_0_ clicks. Otherwise *D*_1_ clicks.

#### Security check

Bob announces his orders of operations. He asks all participants to publish their measurement results of the A photon (signal “0” or “1”). Bob calculates the error probability *P*_*eT*_ and compares it with the average measurement error Γ (see Supplementary I and II), which is caused by environmental noise and implement imperfection. If *P*_*eT*_ is larger, Bob terminates the communication. If there is no security problem being found, the number B becomes the shared signal. Then, all communicators begin the next round of signal transfer process.

### Message Exchange

After step (2) is repeated many times, a series of random bits are shared by multiple users. The participants can use them as a key to exchange information. What they need to do is to announce which detector clicks for each shared signal. As for Bob, he can also use the same shared key to encode his real message and publish the corresponding ciphertext.

The above is the proposed quantum secure group communication protocol. The basic idea is not to generate a key within many authorized users but to directly distribute a pre-selected key. The pre-selected key is decided by Bob himself and is used only if the communication channel is secure. Next we emphasize five points.

First, the pre-selected key is transferred to all participants independently. Therefore, if a transmission channel between Bob and one participant is not secure, Bob can simply cut it off by *SD* (i.e., Bob blocks that participant’s photons), which does not affect the communication between him and others. Moreover, if Bob’s phase operation is fast enough (during *T*_*s*_, he is able to send different participants different signals), he can group participants and make different groups have different authorizations. He only sends the complete key to the users with the highest authorization while he sends the less privileged users only part of the key (by blocking some signals). Then, those less privileged users cannot get all the information in the message exchange stage.

Second, like usual QKD protocols, our protocol is also susceptible to the photon-number-splitting (PNS)^52^ attacks when weak coherent pulses are used. To defend PNS attacks, we can use decoy state technologies^14,18–20^ which is widely implemented in practical QKD systems. When weak coherent pulses are utilized, according to our protocol, each participant’s coherent pulse passes the transmission channel twice. The first time is from the participant to Bob while the second time is from Bob to the participant. We notice that Eve cannot extract Bob’s information if she implements PNS attacks only when the participant’s photon travels from Bob to the participant. However, if Eve attacks when the photon travels from the participant to Bob, she can get the time window of SD. Then, Eve can make her photon into Bob’s station and bring back the information of Bob’s phase operation. Therefore, we must secure the transmission channel when the participant’s photon travels from the participant to Bob. Since Eve doesn’t know when participant’s photons pass through the transmission channel, the participant can insert decoy states which are used to detect Eve’s PNS attacks, while detections are achieved by SD. Then, during the security check, Bob and participants can analyze whether there are PNS attacks.

Third, due to *OD*_2_ and the short time window Δ*t*, the counterfactual quantum attack is defeated since it can not be completed in time.

Fourth, we adopt the same strategy as the Ping-Pong protocol to ensure communication security, i.e., we check the path entanglement of participant’s photons. Those A photons correspond to control mode in the Ping-Pong protocol while B photons correspond to message mode. However, since path entanglement is utilized here, if Bob directly does the measurement, he only has half the chance to find photons. It is not efficient and the result is confused with that of photon loss. Therefore, the measurement in our security check process is done mainly by participants rather than Bob.

Fifth, we check the security for each Bob’s signal, since one Bob’s signal is measured by many participants. We notice that Eve can randomly intercept some participants’ photons to get the information of the time window so that she can steal Bob’s information. In fact, this happens in all network communications, as long as Bob sends the same message to many users. For example, let us consider a secure communication network based on QKD. Eve can eavesdrop small fragments of a key from different participants. Each fragment can help Eve to read a short piece of Bob’s information. Moreover, supposing Eve gets a fragment of the key from one communicator, such as Alice, she can not only read Bob’s corresponding message but also utilizes the message to decode other communicators’ keys such as the key shared by Bob and Sam. Then, Eve also gets a piece of Sam’s information. Therefore, although in the secure network based on QKD, every two communicators have a unique key, the information they exchanged can still be regarded as encrypted by Bob’s message. Hence, why don’t we skip the intermediate steps and just transfer a determined key? Does the secure network based on QKD have some advantages? In the next section, we will analyze and discuss that.

## Discussion on Network Communication Security and Efficiency

Suppose that Eve hacks *m* participants for one Bob’s signal while for each participant, she intercepts *k*(*k* = 1, 2) photons. In our protocol, if Eve intercepts a “B” photon, she does not have to accept the security check. Apparently, Eve has *P*_*B*_ = 50% probability of capturing the photon. When that happens, Eve knows exactly when *SD* is turned off. Then, Eve can send her own photon into Bob’s device and get Bob’s signal for 100%. Thus, the probability of Eve stealing Bob’s signal is *P*_*B*_ = 50%. In contrary, if Eve intercepts an “A” photon, she will be checked and she has no chance to read Bob’s real signal. We notice once Eve measures a participant’s photon, the photon entanglement is destroyed no matter whether Eve captures the photon or not. Even if Eve’s detector gets nothing, the participant’s detectors still have 50% chance clicking incorrectly, which exposures Eve. If Eve’s detector clicks, it means there is no photon at the participant’s end, which helps to expose Eve. To reduce the chance of being exposed, Eve can return a fake photon to the participant, which causes the wrong participant’s detector to click for 50%. Therefore, if Eve intercepts an “A” photon, the chance of her exposure is *P*_*A*_ = 50%. Thus, the total chance of Eve getting Bob’s one signal from one participant without exposure is

2 $${P}_{s}={\mathrm{(1}-{P}_{A})}^{k-1}{P}_{B}\frac{{C}_{1}^{k-1}}{{C}_{2}^{k}}.$$

We notice that (1 − *P*_*s*_)^*m*^ represents the chance that either Eve does not know Bob’s signal or she is exposed after she attacks *m* participants. Then, the total chance of her stealing Bob’s signal without exposure is

3 $${P}_{sT}=1-{\mathrm{(1}-{P}_{s})}^{m}.$$

Here, it is easy to see that *P*_*sT*_ = 1 − (3/4)^*m*^ for both *k* = 1 and *k* = 2.

In addition, the total probability of Eve being exposed after Bob checks *n* “A” photons is

4 $${P}_{eT}=\frac{km}{2n}{P}_{A}.$$

Above, we assume that the communication is free from noise and implement imperfection. In practical application, Eve will not be exposed if *P*_*eT*_ is smaller than the average measurement error (Γ) due to environmental noise and implement imperfection. According to Eq. (4), as *n* increases, the probability of Eve being found is getting smaller. It indicates the network communication requires higher error control in order to reduce the risk of eavesdropping. As for Eve, she needs to minimize *m* in order to reduce the risk of being exposed. However, if she does so, it also reduces the chance of her stealing Bob’s information according to Eq. (3).

Next, we consider a secure quantum network based on BB84. In the communication, Bob generates *n* − 1 independent keys with *n* − 1 participants so that they can exchange information using those keys. In the process of generating a key, one participant selects either the computational basis or the Hadamard basis to encode a bit while Bob randomly selects one of those two bases to infer the bit. As long as their selections are the same, the bit is shared by the participant and Bob. Otherwise, Bob’s measurement result is meaningless and can be discarded. It is easy to see that the key generation probability is 1/2. In addition, for security reasons, Bob and the participant need to ensure the consistency of their shared bits.

Based on the above discussion, in the next analysis of eavesdropping, we only consider the case when Bob and the participant announce the identical basis. In the meantime, we assume that Bob checks one of every two bits with the participant. The detailed model is as follows. One participant launches four photons to Bob. On average, only two of them can be utilized to generate the key. Bob randomly selects one of these two photons to check the security. This photon corresponds to the “A” photon in our protocol. Then, the remaining photon is the key, which corresponds to the “B” photon in our protocol. Here we still assume that Eve hacks *m* participants and intercepts *k* of one participant’s two photons. She measures each photon by one random basis. According to her measurement result, she sends a fake photon to Bob. If Eve captures the “B” photon, apparently, she has 50% chance of choosing the correct basis (Notice that Bob and the participant’s bases are the same). Then, Eve gets the key certainly. The probability of Eve stealing Bob’s bit without exposure is ${P^{\prime} }_{B}=\mathrm{50 \% }$. Next we consider the situation that Eve captures the “A” photon. Apparently, she will not be exposed if she selects the correct basis. However, if Eve selects a wrong basis, she has 50% chance being exposed. As a result, the probability of Eve being exposed is ${P^{\prime} }_{A}=\mathrm{25 \% }$. Then, the total chance of her stealing Bob’s signal without exposure is

5 $${P^{\prime} }_{sT}=1-{[1-{(1-{P^{\prime} }_{A})}^{k-1}{P^{\prime} }_{B}\frac{{C}_{1}^{k-1}}{{C}_{2}^{k}}]}^{m}\ge {P}_{sT}.$$

Here we can see that if *k* = 1, ${P^{\prime} }_{sT}=1-{\mathrm{(3/4)}}^{m}$ while if *k* = 2, ${P^{\prime} }_{sT}=1-{\mathrm{(5/8)}}^{m}$. In addition, the total probability of Eve being exposed after Bob checks *n* “A” photons is

6 $${P^{\prime} }_{eT}=\frac{km}{2n}{P^{\prime} }_{A} < {P}_{eT}.$$

Comparing the results of the above two scenarios, we can see that our proposed protocol is safer and more efficient. The main difference comes from *P*_*A*_. In our protocol, Eve has 50% chance of exposure when she measures the “A” photon, but in the network based on BB84, the probability is 25%. This is determined by the nature of the QKD protocol. The shared random bit is generated during the communication. If Eve happens to choose the right operation, she will not leave any abnormal trace. However, in our protocol, the random bit is pre-prepared before the communication. It is delivered certainly and directly to all participants. Once Eve interferes with the delivery process, she immediately creates a traceable error. In addition to the enhancement of the security, we should also mention that the direct signal delivery process improves the key generation probability. Our protocol only needs two photons to generate a key while in the network based on BB84, four photons generate one key.

## Conclusion

In summary, we report a new kind of secure quantum group to group communication protocol. A “shared” key is securely transferred to all group members so that they can use it to encode and decode their messages. By changing the phase at one arm of one participant’s interferometer, Bob can exactly control which detector of the participant to be clicking. Based on that, Bob can directly send a pre-selected key to all participants. In the meantime, a quantum multi-user authorization system is applied to give authorization to all participants in the group. It secures the key transfer processes. The main principle of protection is due to the fact that Eve can only access one arm of every participant’s interferometer. Any attempt that she tries to measure one participant’s photon simply destroys the interference, which causes errors in participant’s measurement and shows her presence. Moreover, we show the quantum multi-user authorization system can defeat counterfactual quantum attack. Counterfactual quantum attack tries to steal information by an untraceable photon. It is very hard to be exposed. However, the counterfactual quantum attack requires a photon being operated by Bob more than once (consistent operation). Therefore, we precisely control the communication time so that Eve can not complete the attack in time. As a result, we can share secure messages among a large number of users. At the end of the paper, we present the advantage of our protocol by comparing our protocol with the quantum secure network based on BB84. We show that our protocol is more efficient and securer since the key is transferred directly.

## Electronic supplementary material

Supplementary materials
