# Supplementary material for: Quantum Secure Group Communication
Source: Sci Rep. 2018 Mar 1;8:3899. doi: 10.1038/s41598-018-21743-w (PMC5832868; doi:10.1038/s41598-018-21743-w)
Supplement: Supplementary file 1 — Supplementary materials [file 41598_2018_21743_MOESM1_ESM.pdf]

Supplementary materials: Quantum Secure Group Communication

Zheng-Hong Li[1,+,*], M. Suhail Zubairy[2,+] & M. Al-Amri[2,3,4,+]

[1]Department of Physics, Shanghai University, Shanghai 200444, China

[2]Institute for Quantum Science and Engineering (IQSE) and Department of Physics and Astronomy, Texas A&M University, College Station, Texas 77843-4242, USA

[3]The National Center for Applied Physics, KACST, P.O.Box 6086, Riyadh 11442, Saudi Arabia

[4]Department of Physics, KKU, P.O. Box 9004, Abha 61413, Saudi Arabia

*The corresponding author: refirefox@shu.edu.cn

[+]These authors contributed equally to this work

I. The influence of the imperfection of the beam-splitter

Here we briefly discuss the influence of the imperfection of the BS in the communication system. Apparently, if the reflectivities of BS s are not exactly 50%, then neither D_0 nor D_1 clicks with unit probability, which causes communication error. In the following discussion, we set the reflectivity of the BS at participant's end as $\cos^2\theta_1$, while the reflectivity of the BS at Bob's end is $\cos^2\theta_2$. Accordingly, the function of the BS, i.e., the Eq. (1), can be rewritten as

$$\begin{aligned} |P0\rangle_u &\to \cos\theta_u |P0\rangle_u + \sin\theta_u |0P\rangle_u \\ |0P\rangle_u &\to -\cos\theta_u |0P\rangle_u + \sin\theta_u |P0\rangle_u \end{aligned}, \tag{A1}$$

where $u = 1, 2$. In Eq. (A1), $|P0\rangle_1$ represents the participant's photon is in the private path of the participant's interferometer; $|0P\rangle_1$ represents the photon is in the public path but not inside Bob's device; $|P0\rangle_2$ represents the photon is inside Bob's device and passes through the red dashed path; $|0P\rangle_2$ represents the photon is inside Bob's device and passes through the blue dotted path.

In the communication, at one participant's end, once a photon, whose initial state is $|P0\rangle_1$, passes through BS_1, the photon state becomes

$$|P0\rangle_1 \to \cos\theta_1 |P0\rangle_1 + \sin\theta_1 |0P\rangle_1 . \tag{A2}$$

Then, the photon with state $\left|0P\right\rangle_1$ goes into Bob's device. According to Bob's different operations, it will be sent into either the red dashed path or the blue dotted path. Utilizing Eq. (A1), the corresponding outputs are

$$\begin{aligned} \left|P0\right\rangle_2 &\rightarrow \sin 2\theta_2 \left|P0\right\rangle_2 - \cos 2\theta_2 \left|0P\right\rangle_2 \\ \left|0P\right\rangle_2 &\rightarrow -\cos 2\theta_2 \left|P0\right\rangle_2 - \sin 2\theta_2 \left|0P\right\rangle_2 \end{aligned} . \quad \text{(A3)}$$

As we have indicated in the main text, under ideal conditions, the photon gets out Bob's interferometer from the same entrance where it enters the interferometer. However, if Bob's BS is not perfect, the photon may go the wrong way and cause D_2 clicking. It means the photon never returns to its participant. Measurement error occurs. As shown in Eq. (A3), the probability of D_2 clicking is $\cos^2 2\theta_2$ no matter what Bob's operation is. In the meantime, we notice that the participant's photon has the probability of $\sin^2\theta_1$ appearing at Bob's end. Therefore, the total probability of D_2 clicking is

$$P_{D2} = \sin^2\theta_1 \cos^2 2\theta_2 . \quad \text{(A4)}$$

Correspondingly, the probability of the photon not being blocked by Bob and returning to the participant is $\sin^2\theta_1 \cos^2 2\theta_2$.

Now we consider the probabilities of participant's detectors clicking. It is not difficult to find out that, if the returned photon component does not have a phase change, the finial photon state is

$$\begin{aligned} \left|P0\right\rangle_1 \xrightarrow{\text{no phase change}} & \left(\cos^2\theta_1 + \sin^2\theta_1 \sin 2\theta_2\right)\left|P0\right\rangle_1 \\ & + \cos\theta_1 \sin\theta_1 \left(1 - \sin 2\theta_2\right)\left|0P\right\rangle_1 \end{aligned} . \quad \text{(A5)}$$

It should only cause D_1 clicking under ideal conditions. However, here the probability of D_1 clicking is

$$P_{D1} = \left|\cos^2\theta_1 + \sin^2\theta_1 \sin 2\theta_2\right|^2 . \quad \text{(A6)}$$

In contrast, if the returned photon component has a π phase change, the finial photon state is

$$\begin{aligned} \left|P0\right\rangle_1 \xrightarrow{\pi\ \text{phase change}} & \left(\cos^2\theta_1 - \sin^2\theta_1 \sin 2\theta_2\right)\left|P0\right\rangle_1 \\ & + \cos\theta_1 \sin\theta_1 \left(1 + \sin 2\theta_2\right)\left|0P\right\rangle_1 \end{aligned} . \quad \text{(A7)}$$

The probability of D_0 clicking is

$$P_{D0}=\left|\cos\theta_1\sin\theta_1\left(1+\sin 2\theta_2\right)\right|^2. \tag{A8}$$

Then, the average measurement error due to the imperfection of the BS can be defined as

$$\Gamma=\max\{1-P_{D0},1-P_{D1}\}. \tag{A9}$$

As an example, next we assume that $\theta_1=\theta_2=\pi/4+\Delta$ where Δ is very small. After neglecting third and higher order contributions in $|\Delta|$, we have $P_{D2}\approx 2|\Delta|^2$, $P_{D1}\approx 1-2|\Delta|^2$ and $P_{D0}\approx 1-6|\Delta|^2$. Therefore, we can get that $\Gamma=6|\Delta|^2$.

II. The influence of the imperfection of the transmission channel

Next we discuss the influence of the imperfection of the transmission channel. We assume that the maximum dissipation of the public path of a participant's interferometer (The path length is equal to the distance between the participant and Bob) is κ_{01} while the maximum dissipation of the private path is κ_{10}. In addition, the maximum phase difference between two paths is φ. Then, after a photon passes the transmission channel, the influence of the dissipation and the phase noise can be described as[39]

$$\begin{aligned}&\left|P0\right\rangle_1\rightarrow\sqrt{1-\kappa_{10}}\left|P0\right\rangle_1\\&\left|0P\right\rangle_1\rightarrow e^{i\varphi}\sqrt{1-\kappa_{01}}\left|P0\right\rangle_1\end{aligned}. \tag{B1}$$

For the convenience of discussion, we suppose that the phase difference due to different Bob's operations is exactly π. This can be tested and adjusted before the communication starts. Moreover, we assume all optical elements such as BS s at Bob's and participants' stations are perfect (In fact, their influence can be included in parameters κ_{10}, κ_{01} and φ). Then, according to Eq. (A2) in Supplementary I, when a participant's photon reaches Bob's station, the photon state can be expressed as ($\theta_1=\pi/4$)

$$\left|P0\right\rangle_1\rightarrow\frac{\sqrt{2}}{2}\left(\sqrt{1-\kappa_{10}}\left|P0\right\rangle_1+e^{i\varphi}\sqrt{1-\kappa_{01}}\left|0P\right\rangle_1\right). \tag{B2}$$

Next Bob manipulates the photon phase. According to Bob's different operations, the photon states can be described as $\sqrt{2}\left(\sqrt{1-\kappa_{10}}\left|P0\right\rangle_1\pm e^{i\varphi}\sqrt{1-\kappa_{01}}\left|0P\right\rangle_1\right)/2$, where "+" means that Bob doesn't change the photon phase while "−" means that Bob adds a π phase shift. After Bob's operation, the photon is sent back to the participant. It passes the transmission channel again and its state becomes

$\sqrt{2}\left[\left(1-\kappa_{10}\right)\left|P0\right\rangle_1 \pm e^{i2\varphi}\left(1-\kappa_{01}\right)\left|0P\right\rangle_1\right]/2$. Then, the photon passes through participant's BS. According to Eq. (1) in the paper, the state of the photon measured by the participant is

$$\left|F_{\pm}\right\rangle=\frac{1}{2}\left\{\left[\left(1-\kappa_{10}\right)\pm\left(1-\kappa_{01}\right)e^{i2\varphi}\right]\left|P0\right\rangle_1+\left[\left(1-\kappa_{10}\right)\mp\left(1-\kappa_{01}\right)e^{i2\varphi}\right]\left|0P\right\rangle_1\right\}. \quad \text{(B3)}$$

We notice that if Bob doesn't change the photon phase, participant's detector D_1 should click ($\left|P0\right\rangle_1$), while if Bob adds a π phase shift, participant's detector D_0 should click ($\left|0P\right\rangle_1$). It is easy to see that the probabilities of those two detectors clicking correctly are the same, which are $\left|\left[\left(1-\kappa_{10}\right)+e^{i2\varphi}\left(1-\kappa_{01}\right)\right]\right|^2/4$. Then, the measurement error Γ can be defined as

$$\begin{aligned}\Gamma&=1-\left|\frac{1}{2}\left[\left(1-\kappa_{10}\right)+e^{i2\varphi}\left(1-\kappa_{01}\right)\right]\right|^2\\&=1-\frac{1}{4}\left[2\left(1+\cos 2\varphi\right)\left(1-\kappa_{10}-\kappa_{01}\right)+\kappa_{10}^2+\kappa_{01}^2+2\cos 2\varphi\kappa_{10}\kappa_{01}\right].\\&\approx\kappa_{10}+\kappa_{01}+\varphi^2\end{aligned} \quad \text{(B4)}$$

In the approximation, we assume κ_{10}, κ_{01} and φ are very small. We then utilize $\cos\varphi\approx 1-\varphi^2/2$ and neglect terms including $\kappa_{10(01)}^2$, $\kappa_{10}\kappa_{01}$ and $\varphi^2(\kappa_{10}+\kappa_{01})$. As discussed in the paper, Γ should be smaller than the total probability of Eve being exposed (P_{eT}) which is given in Eq. (4), otherwise the communication is not secure.

The above discussion is based on the fact that the participant does not change his measurement basis. However, it is not difficult to see that

$$\left\langle F_+\middle|F_-\right\rangle=\frac{1}{2}\left(\kappa_{01}-\kappa_{10}\right)\left[2-\left(\kappa_{01}+\kappa_{10}\right)\right]. \quad \text{(B5)}$$

The orthogonality of the two photon states according to Bob's two operations is irrelevant to the phase noise.

III. Does successful single-cycle counterfactual quantum attack exist?

In the main text, we claim that a counterfactual quantum attack cannot be completed if Bob only preforms his operation on Eve's photon once. Here, we give the proof.

Suppose Eve's photon is in a superposition state which is composed of orthogonal states $\left\{\left|E_i\right\rangle,\left|B_j\right\rangle\right\}(i,j=0,1,2,....)$, where states $\left\{\left|B_j\right\rangle\right\}$ constitute a subsystem which can be affected by Bob. In our case, they represent the possible states of the photon leaking into the

transmission channel. In contrast, states $\{|E_i\rangle\}$ mean that the photon is in Eve's device. We assume Bob cannot affect $|E_i\rangle$, then we have

$$\begin{cases} O_\kappa |E_i\rangle = |E_i\rangle \\ O_\kappa |B_{j'}\rangle = \sum_j \beta_{\kappa j} |B_j\rangle \end{cases}. \tag{C1}$$

Here $O_\kappa(\kappa = 0,1,2,....)$ represents Bob's unitary operation, which satisfies $O_\kappa^\dagger O_\kappa = I$ with unitary matrix I. Then, the amplitude $\beta_{\kappa j}$ satisfies $\sum_j |\beta_{\kappa j}|^2 = 1$.

Now we consider the case when Eve tries to measure Bob's operation. We assume Eve's initial photon state is

$$|\psi\rangle = \sum_i a_i |E_i\rangle + b_0 |B_0\rangle \tag{C2}$$

In the measurement, Eve's photon will be only operated by Bob once. In addition, since Eve wants to achieve an untraceable measurement, the probability of the photon appearing at Bob's end, i.e., $|b_0|^2$, should be close to zero.

After Bob's operation, Eve's photon state becomes

$$|\psi_\kappa\rangle = O_\kappa |\psi\rangle = \sum_i a_i |E_i\rangle + b_0 O_\kappa |B_0\rangle. \tag{C3}$$

Then, corresponding to two different Bob's operations O_κ and $O_{\kappa'}$, the inner product of $|\psi_\kappa\rangle$ and $|\psi_{\kappa'}\rangle$ is

$$\begin{aligned} \langle \psi_\kappa | \psi_{\kappa'} \rangle &= \sum_i |a_i|^2 + |b_0|^2 \langle B_0 | O_\kappa^\dagger O_{\kappa'} | B_0 \rangle \\ &= \sum_i |a_i|^2 + |b_0|^2 \beta_{\kappa\kappa' 0} \end{aligned}. \tag{C4}$$

For the above calculation, we emphasize two points. Firstly, according to Eq. (C1), we have $\langle B_0 | O_\kappa^\dagger | E_{i'} \rangle = \langle E_i | O_{\kappa'} | B_0 \rangle = 0$. Secondly, $O_\kappa^\dagger O_{\kappa'}$ can be regarded as one operation performed by Bob. Then, we have $\langle B_0 | O_\kappa^\dagger O_{\kappa'} | B_0 \rangle = \langle B_0 | \left(\sum_j \beta_{\kappa\kappa' j} | B_j \rangle \right) = \beta_{\kappa\kappa' 0}$, where $|\beta_{\kappa\kappa' 0}| \le 1$.

We notice that only if $\langle \psi_\kappa | \psi_{\kappa'} \rangle = 0$, Eve can distinguish Bob's two operations. Then, we can get the following condition

$$\sum_i |a_i|^2 + |b_0|^2 \beta_{\kappa\kappa' 0} = 0. \tag{C5}$$

However, since $|b_0|^2$ should be close to zero while $|\beta_{\kappa\kappa' 0}| \le 1$, the condition Eq. (C5)

cannot be satisfied. Consequently, it is impossible for Eve to steal Bob's information by an untraceable way if Bob only operates her photon once.

Above we show that a successful single-cycle counterfactual quantum attack does not exist. Nonetheless, if Bob can operate Eve's photon more than once, the conclusion is different. In this case, we need also to consider the influence of Eve's operation (O_E) which can affect all her possible photon states and satisfies $O_E^\dagger O_E = I$ (Therefore, it has no contribution in the single-cycle case).

If Eve operates her photon between every two operations of Bob, then after M operating cycles, Eve photon state is

$$\left|\psi_\kappa\right\rangle = \left(O_E O_\kappa\right)^M \left|\psi\right\rangle. \tag{C6}$$

If $[O_E, O_\kappa] \neq 0$, it is possible to satisfy $\left\langle\psi_\kappa \middle| \psi_{\kappa'}\right\rangle = 0$ as shown in Ref.[35].
